# Prognostic Value of Platelet-to-Lymphocyte Ratio in Non-Muscle Invasive Bladder Cancer Patients: Intravesical Bacillus Calmette-Guerin Treatment After Transurethral Resection of Bladder Tumor

**DOI:** 10.3389/fsurg.2022.907485

**Published:** 2022-05-23

**Authors:** Ruicheng Wu, Dengxiong Li, Facai Zhang, Yunjin Bai, Xiaoming Wang, Ping Han

**Affiliations:** Department of Urology, Institute of Urology, West China Hospital, Sichuan University, Chengdu, China

**Keywords:** Bacillus Calmet-Guiren immunotherapy, bladder cancer, platelet-tolymphocyte ratio, progression, non-muscle invasive bladder cancer

## Abstract

The objective of this study was to investigate the platelet-to-lymphocyte ratio (PLR) in patients who underwent intravesical treatment for non-muscle invasive bladder cancer (NMIBC). A total of 197 patients who underwent intravesical Bacillus Calmette-Guerin treatment after transurethral resection of bladder (TURB) were included. We divided the patients into different groups according to the treatment stage before and during induction treatment as Group 1 and Group 2, and set the change value of PLR as the Group 3. The cutoff values of PLR were determined through receiver operation characteristics curves analysis. we found a significant difference in recurrence-free survival (RFS) and progression-free survival (PFS) between patients with high serum PLR and those with low serum PLR in Group 1, as well as Group 2. Cox multivariate analysis revealed that tumor number ≥3, high grade, and history of carcinoma in situ (CIS) were significant factors predicting RFS and PFS. The PLR values before and during induction therapy could be used as predictors for the progression and recurrence of NMIBC patients receiving BCG immunotherapy. the PLR values after induction therapy have a stronger predictive power.

## Introduction

Bladder cancer (BCa) is the tenth most common cancer globally, with approximately 530,000 new cases and 200,000 deaths worldwide each year (1). Approximately 70% of patients are initially diagnosed with non-muscle invasive bladder cancer (NMIBC) (2). Previous study indicated that the 5-year recurrence and progression rates of NMIBC patients ranged from 50% to 70% and from 10% to 30%, respectively. Patients were divided into low-, intermediate-, and high-risk groups according to their clinical and pathological characteristics (3). For intermediate- and high-risk patients, intravesical bacillus Calmette-Guerin (BCG) after transurethral resection of bladder (TURB) is more effective than TURB alone or combination with and intravesical chemotherapy for patients with intermediate and high-risk tumors (4–6).

Despite considerable progress in BCG intravesical treatment, approximately 20%–40% of patients still do not benefit from treatment (7). In addition, BCG instillations included induction therapy (8) and maintenance therapy (9). Several patients in high-risk group may progress to muscle invasive bladder cancer (MIBC) during the treatment period, Then had worse prognosis than those who present with primary muscle-invasive disease (10). Therefore, some studies suggested that radical cystectomy (RC) should be performed as soon as possible for NMIBC patients with a higher risk of progression (11). Such a more aggressive approach must be weighed against its risk, progression rate, and impact on quality of life. In order to achieve high-quality management, it is very important to find the prognostic factors of NMIBC. Several prognostic models based on the clinicopathological features have been proposed to guide the management and follow-up of NMIBC patients (12–15). After external validation with a new dataset, it was found that these models showed poor discrimination between disease recurrence and progression in NMIBC patients. These models overestimate the risk of disease recurrence and progression in high-risk patients (16), promoting urologists to find more accurate biomarkers to improve these models.

The role of the immune system and inflammatory responses in cancer has been increasingly studied. In the tumor microenvironment, the inflammatory cell infiltration associates with prognosis in many solid tumors (17). The systemic inflammatory state could be reflected by circulating inflammatory biomarkers such as neutrophil-to-lymphocyte ratio (18), lymphocyte-to-monocyte ratio (19) and platelet-to-lymphocyte ratio(PLR) (20). A study showed that high NLR before TURB is an independent predictor of NMIBC progression and recurrence (21). Recent researches have reported the prognostic value of PLR in solid tumors (22–24). Taken together, it is tempting to speculate that PLR could contribute to a deeper understanding of the risk stratification of NMIBC and guide clinical practice. In this study, we aimed to evaluate the prognostic value of PLR in NMIBC patients receiving intravesical BCG therapy before and during induction phases.

## Material and Methods

### Patients and Treatment

The study was conducted in accordance with the Declaration of Helsinki (as revised in 2013). The study and disclaimer of informed consent were approved by the West China Hospital of Sichuan University Biomedical Research Ethics Committee (No: 20201071). we retrospectively enrolled the medical records of 362 consecutive NMIBC patients undergoing TURB. Repeat TURB was not routinely performed. Induction BCG instillation was administered according to an empirical 6-week schedule described by Morales et al. (8). Patients would then receive maintenance therapy for at least one year, and the infusion dose was 120 mg. Inclusion criteria were as follows: complete data regarding all pathology and serological reports, complete follow-up cystoscopy and completely received induction therapy and successfully entered the maintenance perfusion therapy phase. In addition, exclusion criteria were: no complete clinical or follow-up data, suffering from urinary tract infection or other chronic infectious diseases, suffering from malignant tumors in other parts, unable to tolerate BCG and receiving infusion of other drugs and history of radiation or hematological disorders. Ultimately, 197 patients were included in this study. The following data were collected by medical records review: age, sex, body mass index (BMI), smoking status, history of gross hematuria, history of hypertension and diabetes, tumor size, tumor lesions, tumor grade, CIS, tumor stage, tumor histology, recurrence-free survival (RFS), progression-free survival (PFS) and PLR before and during induction therapy with BCG instillation. We collected the serological results of patients one week before induction therapy, which was defined as Group 1, and the serological results of patients during induction therapy, which were defined as Group 2. In addition, we included the changes in the PLR values. The PLR change is the ratio of PLR before and during induction therapy, which was defined as group 3. If the result was ≥1, then PLR change was defined as increased; otherwise, it was defined as decreased.

### Follow-up and Outcomes

The postoperative follow-up included physical examination, urine cytology, and cystoscopy scheduled generally every 3 months in the first 2 years, every 6 months for the third year, and once a year afterward. Disease recurrence was defined as the first pathologically proven tumor regardless of stage or grade, whereas disease progression was defined as a pathological diagnosis of muscle-invasive bladder cancer during follow-up.

### Pathological Evaluation

All specimens were assessed by genitourinary pathologists in the West China Hospital, Sichuan University using the TNM staging system of the 2009 American Joint Committee and the 2004/2016 World Health Organization grading system.

## Statistical Analysis

Comparisons among study groups were assessed by Chi-square and Mann–Whitney U tests. Kaplan–Meier curves and the log-rank test were used to estimate and determine the significant differences between study groups. Univariate and multivariate Cox regression analyses were used to test the association between PLR and oncological outcomes before and during induction therapy. we used the Shapiro-Wilk test to evaluate the normal distribution of continuous variables, and neither PFS nor RFS conformed to the normal distribution. The results were considered significant if the two-sided *p* value was <0.05. Data analyses were performed using R 3.6.3 software.

## Results

### Patient Baseline and Prognosis

The clinicopathological characteristics of 197 NMIBC patients are shown in Table 1. The mean age was 64.17 years (standard deviation, SD:11.07) including 170 males and 27 females, and the median follow-up time was 24.68 months (interquartile range, IQR: 14.00–29.00 months). In this cohort, 57 patients had a smoking history and 151 patients (76.6%) had gross hematuria during the disease. A total of 111 patients had multifocal tumor, and 15 patients had CIS. The tumor size of 87 patients was equal to or larger than 3 cm, and 26 patients had pathological variants. Patients classified as tumor progression were also accompanied by recurrence, with a total of 30 patients (15.2%) with recurrence alone and no progression, and a total of 55 patients (27.9%) with recurrence and progression. Patients who discontinued BCG infusion due to side effects but did not subsequently receive other drug infusions were also included.

**Table 1:** Demographic and clinical data of patients in analysis.

	Total	Group1			Group2			Group3		
	197	Low (101)	High (96)	*p* value	Low (83)	High (114)	*p* value	Low (117)	High (80)	*p* value
Sex				0.578			0.104			0.299
Female	27 (13.7%)	12 (6.1%)	15 (7.6%)		7 (3.6%)	20 (10.2%)		19 (9.6%)	8 (4.1%)	
Male	170 (86.3%)	89 (45.2%)	81 (41.1%)		76 (38.6%)	94 (47.7%)		98 (49.7%)	72 (36.5%)	
Age	64.17±11.05	64.89±10.76	63.41±11.41	0.348	65.02±11.14	63.54±11.04	0.356	63.91±10.89	64.55±11.41	0.690
BMI	23.65±3.04	23.75±3.16	23.54±2.94	0.641	23.9±2.68	23.46±3.29	0.323	23.53±2.87	23.82±3.29	0.521
Smoker				0.820			0.637			0.836
No	140 (71.1%)	73 (37.1%)	67 (34%)		57 (28.9%)	83 (42.1%)		82 (41.6%)	58 (29.4%)	
Yes	57 (28.9%)	28 (14.2%)	29 (14.7%)		26 (13.2%)	31 (15.7%)		35 (17.8%)	22 (11.2%)	
Gross hematuria			1.000						0.455	
No	46 (23.4%)	24 (12.2%)	22 (11.2%)		20 (10.2%)	26 (13.2%)		30 (15.2%)	16 (8.1%)	
Yes	151 (76.6%)	77 (39.1%)	74 (37.6%)		63 (32%)	88 (44.7%)		87 (44.2%)	64 (32.5%)	
Hypertension			0.035						0.164	
No	140 (71.1%)	79 (40.1%)	61 (31%)		63 (32%)	77 (39.1%)		88 (44.7%)	52 (26.4%)	
Yes	57 (28.9%)	22 (11.2%)	35 (17.8%)		20 (10.2%)	37 (18.8%)		29 (14.7%)	28 (14.2%)	
Diabetes				0.770			0.654			0.248
No	172 (87.3%)	87 (44.2%)	85 (43.1%)		74 (37.6%)	98 (49.7%)		99 (50.3%)	73 (37.1%)	
Yes	25 (12.7%)	14 (7.1%)	11 (5.6%)		9 (4.6%)	16 (8.1%)		18 (9.1%)	7 (3.6%)	
Tumor size			0.586						0.263	
≤3 cm	110 (55.8%)	54 (27.4%)	56 (28.4%)		50 (25.4%)	60 (30.5%)		61 (31%)	49 (24.9%)	
>3 cm	87 (44.2%)	47 (23.9%)	40 (20.3%)		33 (16.8%)	54 (27.4%)		56 (28.4%)	31 (15.7%)	
Tumor number			0.456			0.215			0.027	
Single	86 (43.7%)	41 (20.8%)	45 (22.8%)		41 (20.8%)	45 (22.8%)		43 (21.8%)	43 (21.8%)	
Multiple	111 (56.3%)	60 (30.5%)	51 (25.9%)		42 (21.3%)	69 (35%)		74 (37.6%)	37 (18.8%)	
WHO grade			0.590						0.957	
Low	55 (27.9%)	26 (13.2%)	29 (14.7%)		23 (11.7%)	32 (16.2%)		32 (16.2%)	23 (11.7%)	
High	142 (72.1%)	75 (38.1%)	67 (34%)		60 (30.5%)	82 (41.6%)		85 (43.1%)	57 (28.9%)	
CIS				0.770			0.084			1.000
No	182 (92.4%)	87 (44.2%)	85 (43.1%)		73 (37.1%)	109 (55.3%)		108 (54.8%)	74 (37.6%)	
Yes	15 (7.6%)	14 (7.1%)	11 (5.6%)		10 (5.1%)	5 (2.5%)		9 (4.6%)	6 (3%)	
T stage				0.020			1.000			0.248
Ta	40 (20.3%)	27 (13.7%)	12 (6.1%)		16 (8.1%)	23 (11.7%)		26 (13.2%)	13 (6.6%)	
T1	157 (79.7%)	74 (37.6%)	84 (42.6%)		67 (34%)	91 (46.2%)		91 (46.2%)	67 (34%)	
Histology				0.441			0.535			0.207
PTCC	171 (86.8%)	90 (45.7%)	81 (41.1%)		74 (37.6%)	97 (49.2%)		105 (53.3%)	66 (33.5%)	
HV	26 (13.2%)	11 (5.6%)	15 (7.6%)		9 (4.6%)	17 (8.6%)		12 (6.1%)	14 (7.1%)	
Recurrence			0.009						1.000	
No	112 (56.9%)	67 (34%)	45 (22.8%)		57 (28.9%)	55 (27.9%)		67 (34%)	45 (22.8%)	
Yes	85 (43.1%)	34 (17.3%)	51 (25.9%)		26 (13.2%)	59 (29.9%)		50 (25.4%)	35 (17.8%)	
Progression			0.070						0.957	
No	142 (72.1%)	79 (40.1%)	63 (32%)		69 (35%)	73 (37.1%)		85 (43.1%)	57 (28.9%)	
Yes	55 (27.9%)	22 (11.2%)	33 (16.8%)		14 (7.1%)	41 (20.8%)		32 (16.2%)	23 (11.7%)	
Risk group			0.375			0.861			0.396	
Intermediate	62 (31.4%)	34 (33.7%)	28 (29.2%)		26 (31.3%)	36 (31.6%)		38 (32.5%)	24 (30%)	
High	114 (57.9%)	54 (53.5%)	60 (62.5%)		47 (56.6%)	67 (58.8%)		64 (54.7%)	50 (62.5%)	
Very high	21 (21.6%)	13 (12.9%)	8 (8.3%)		10 (12.0%)	11 (9.6%)		15 (12.8%)	6 (7.5%)	

*CIS, carcinoma in situ.*

**Table 2:** C-index and AUC values for recurrence and progression in group 1 and group 2.

	Group 1		Group 2	
Recurrence		95% CI		95% CI
C-index	0.635	(0.604–0.666)	0.652	(0.620–0.683)
AUC	0.743	(0.674–0.812)	0.737	(0.668–0.807)
Progression				
C-index	0.704	(0.665–0.742)	0.728	(0.692–0.764)
AUC	0.759	(0.681–0.838)	0.783	(0.714–0.852)

*AUC, Area Under Curve; C-index, index of concordance.*

### The Optimal Cutoff Value for PLR

The cutoff values were calculated for serological results one week prior to induction infusion in Group 1 and for serological results during induction therapy in Group 2, usually at the outpatient follow-up after the third induction infusion. The PLR cutoff point was determined by the receiver operating characteristic (ROC) curve analysis using the Youden index (25). For the PFS cohort, the optimal cutoff values before and during induction therapy were 112.7 and 98.5, respectively. For the RFS cohort, the optimal cutoff values before and during induction therapy were 99.8 and 98.5, respectively. Below the PLR cutoff value is defined as low PLR, above the PLR cutoff value is defined as high PLR.

### Recurrence-Free Survival (RFS) and Progression-Free Survival (PFS)

**Figure 1:**
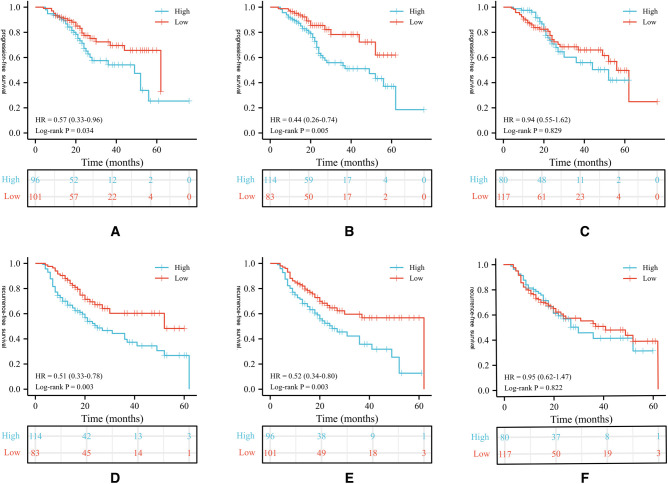
Kaplan-Meier survival curves of oncological outcomes according to PLR values in patients. Survival curves of PLR values for PFS in group 1 (**A**), 2 (**B**), 3 (**C**); Survival curves of PLR values for PFS in group 1 (**D**), 2(**E**), 3(**F**). PLR, platelet-to-lymphocyte ratio; RFS, recurrence-free survival; PFS, progression-free survival.

**Figure 2:**
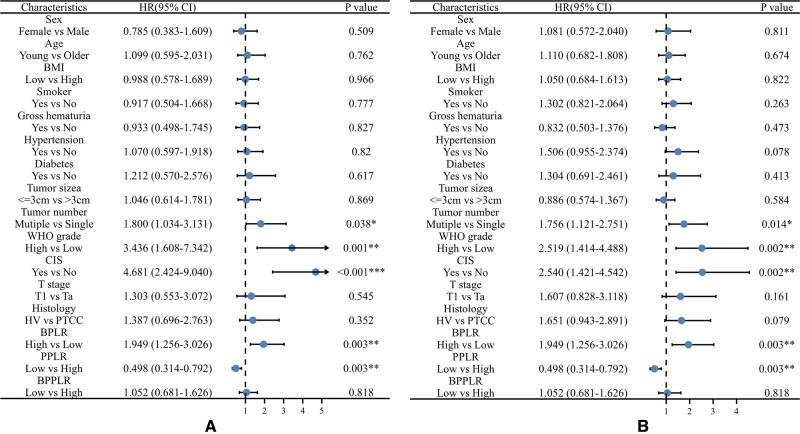
Univariable Cox regression analyses for the prediction of progression-free survival (**A**) and recurrence-free survival (**B**) in patients. BPLR, PLR values of before-induction; PPLR, PLR values of post-induction; BPPLR, the ratio of PLR before and post induction therapy; HV, histology variation; PTCC, Pure urothelial carcinoma; CIS, carcinoma in situ.

**Figure 3:**
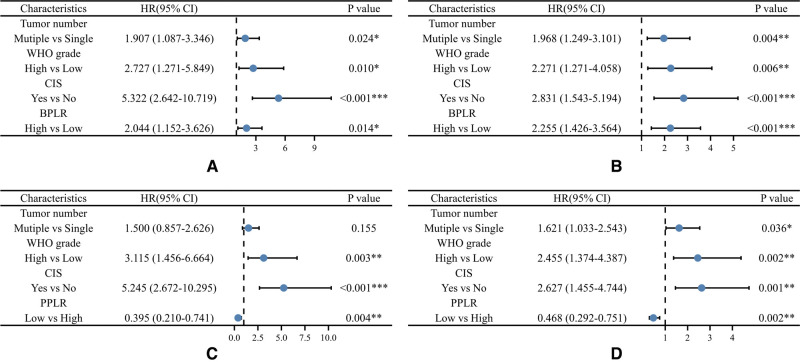
Multivariable Cox regression analyses for the prediction of progression-free survival (**A**) and recurrence-free survival (**B**) in patients. BPLR, PLR values of before-induction; PPLR, PLR values of post-induction; CIS, carcinoma in situ.

During the follow up, 85 (43.1%) patients developed pathological disease recurrence. Kaplan–Meier survival curves showed no significant difference in RFS between patients with increased and decreased PLR change Figure 1. There was no significant association between serum PLR change and the risk of disease recurrence in the univariate analysis (HR:1.052, 95% CI, 0.681–1.626, *p* = 0.018) Figure 2. Conversely, we found a significant difference in PFS and RFS between patients with high serum PLR and those with low serum PLR in Group 1, as well as Group 2. In group 1, high PLR was significantly associated with a higher risk of progression to MIBC (HR: 1.949, 95% CI, 1.256–3.026, *p* = 0.003). In the multivariate analysis, PLR was an independent factor for RFS (HR: 2.255, 95% CI, 1.426–3.564, *p* = 0.001). In Group 2, low PLR was significantly associated with a lower risk of progression to MIBC (HR: 0.498, 95% CI, 0.314–0.792, *p* = 0.001). In multivariate analysis, PLR retained its independent association with RFS (HR: 0.395, 95% CI, 0.210–0.741, *p* = 0.004). In both two groups, tumor number ≥3, high grade, and history of CIS displayed higher risks of tumor progression and recurrence Figure 3.

### Construction of Prognostic Nomograms

**Figure 4:**
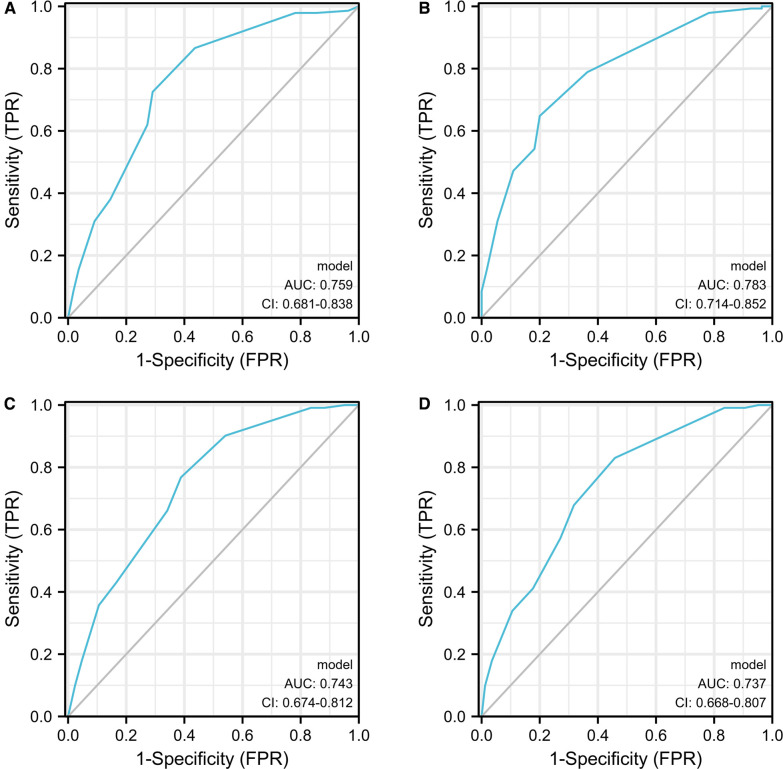
The ROC curves developed for recurrence and progression prediction models. The ROC curve developed for progression prediction model for group 1 (**A**) and group 2 (**B**); The ROC curve developed for recurrence prediction model for group 1 (**C**) and group 2 (**D**).

Nomograms for RFS and PFS was formulated based on the results of the multivariate Cox regression analyses (Supplementary Figure S2). ROC curves of Group 1 predicting PFS were shown in Figure 4, with the AUC 0.743 and 0.759, respectively. Moreover, the C-index also showed predictive accuracy in both RFS and PFS for Group 1 (RFS, C-index 0.635, 95% CI, 0.604–0.666; PFS, C-index: 0.704, 95% CI, 0.665–0.742). Similarly, we found that the nomograms could predict the RFS and PFS of Group 2 effectively (RFS, C-index: 0.652 CI, 0.620–0.683; AUC 0.737, 95% CI, 0.668–0.807; PFS, C-index: 0.728, 95% CI, 0.692–0.764; AUC: 0.783, 95% CI, 0.714–0.852) Table 2.

## Discussion

Many tumor-related factors and patient performance contribute to the recurrence and progression of BCa. In previous studies, the pathological features of tumors were frequently used to predict the prognosis of patients (16). From the mechanical viewpoint, proinflammatory factors in the tumor microenvironment play a key role in tumor growth (26). Systemic inflammation may promote tumor growth by affecting the tumor microenvironment, leading to poor prognosis. And the systemic inflammatory (27) response can be reflected by changes in the number of peripheral blood cell amounts. Platelets can support tumor growth by promoting angiogenesis and producing adhesion molecules (28). Previous Study have shown that platelets can also protect tumor cells in the vasculature from clearance by NK cells (29). In contrast, lymphocytes play a critical role in anti-tumor responses (30). In particular, increased tumor-Infiltrating lymphocytes is associated with a better prognosis for in many different tumor types (31)**.** Therefore, PLR has the potential to be an effective prognostic biomarker.

Previous studies have also discussed whether PLR could be a potential prognostic marker for tumors. Guan et al. reported that PLR had a significant association with an inferior prognosis in metastatic castration-resistant prostate cancer patients (32). Greater PLR also significantly related to poor progress of oral cancer (33). In the research of Liu et al., higher PLR was associated with poor disease-free survival and overall survival (34). Few studies have concentrated on the relationship between PLR and NMIBC patients receiving BCG instillation. In this study, we found that patients with a higher PLR were more prone to BCa recurrence and progression, which was opposite to the previous results conducted by Hyeong et al. 28. We propose the following potential causes. First, we excluded some patients with chronic immune and hematological diseases to avoid confounding factors. Second, ethnic differences might be a possible reason. Finally, the two studies also had different cutoff values, which might affect on patient grouping. A previous meta-analysis showed no significant prognostic value of PLR in BCa patients in terms of RFS (HR = 1.72, 95% CI, 0.79–3.75, *p* = 0.175) (35). Because only two studies were enrolled in the study, the results should be interpreted cautiously.

After BCG instillation, approximately 50% of patients fail to achieve durable responses, and approximately 15% of patients progress to MIBC (36). At least one year of BCG maintenance therapy is required to obtain a significant benefit of BCG in preventing recurrence or progression (37). Previous studies have shown that patients who experience disease progression to MIBC have a worse prognosis (38). Thus, it is important to identify such high-risk patients as soon as possible. For the first time, we showed the critical time point of induction therapy. We found that the high PLR before and during induction therapy could predict a higher risk of recurrence and progression for NMIBC patients. For the management of such patients, more active treatment methods can be considered to save on costs and improve the overall prognosis. In addition, in accordance with a previous study, tumor number ≥3, high grade, a history of CIS were high-risk factors of tumor progression and recurrence (4).

We acknowledge the following limitations. First, our study was a retrospective review of a single institution which is subject to selection and follow-up bias. Second, the timing of PLR measurements was not exactly the same. Finally, we did not use the 1973 WHO grading system, which may reduce comparability with previous studies. Despite these limitations, we first found that PLR before and during induction therapy could be a prognostic factor for progression and recurrence in NMIBC patients treated with BCG-immunotherapy.

## Conclusion

The PLR values before and during induction therapy could be used as predictors for the progression and recurrence of NMIBC patients receiving BCG immunotherapy. The PLR values after induction therapy have a stronger predictive power.

## Data Availability

The raw data supporting the conclusions of this article will be made available by the authors, without undue reservation.
