# Supplementary figures and images for: Prognostic Value of Platelet-to-Lymphocyte Ratio in Non-Muscle Invasive Bladder Cancer Patients: Intravesical Bacillus Calmette-Guerin Treatment After Transurethral Resection of Bladder Tumor
Source: Front Surg. 2022 May 23;9:907485. doi: 10.3389/fsurg.2022.907485 (PMC9411071; doi:10.3389/fsurg.2022.907485)

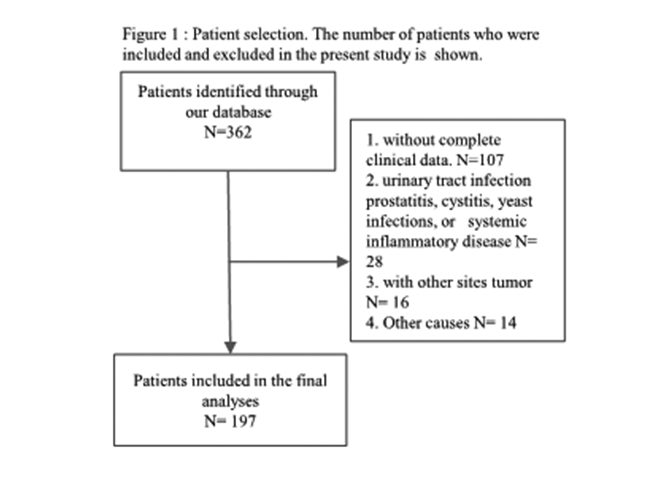

Supplement: Supplementary file 1 [file Image_1_v1.tif]
